# Antibodies against six human herpesviruses in relation to seven cancers in black South Africans: A case control study

**DOI:** 10.1186/1750-9378-1-2

**Published:** 2006-09-14

**Authors:** A Berrington de González, MI Urban, F Sitas, N Blackburn, M Hale, M Patel, P Ruff, R Sur, R Newton, V Beral

**Affiliations:** 1 Cancer Research UK Epidemiology Unit, University of Oxford, Richard Doll Building, Roosevelt Drive, Headington, Oxford, OX3 7LF, UK; 2 Johns Hopkins Bloomberg School of Public Health, 615 North Wolfe Street, Baltimore MD, 21205, USA; 3 MRC/NHLS/Wits Cancer Epidemiology Research Group, National Health Laboratory Service, P.O. Box 1038, Johannesburg 2000, South Africa; 4 The Cancer Council New South Wales, Sydney, Australia; 5 The Schools of Public Health, Universities of New South Wales and Sydney, Australia; 6 National Institute for Communicable Diseases, National Health Laboratory Service, 1 Modderfontein Road, Sandringham 2131, South Africa; 7 Department of Anatomical Pathology, Chris Hani Baragwanath Laboratory, School of Pathology, University of the Witwatersrand, and National Health Laboratory Service, P.O. Box 1038, Johannesburg 2000, South Africa; 8 Clinical Haematology Division, Department of Medicine, Chris Hani Baragwanath Hospital and the University of the Witwatersrand, 7 York Road, Parktown, Johannesburg 2193, South Africa; 9 Division of Medical Oncology, Department of Medicine, Johannesburg Hospital and the University of the Witwatersrand, 7 York Road, Parktown, Johannesburg 2193, South Africa; 10 Division of Radiation Oncology, Department of Medicine, Johannesburg Hospital and the University of the Witwatersrand, 7 York Road, Parktown, Johannesburg 2193, South Africa; 11 Hamilton Regional Cancer Centre, 699 Concession Street, Hamilton, ON L8V 5C2, Canada

## Abstract

**Background:**

Infections with certain human herpesviruses have been established as risk factors for some cancer types. For example, Epstein-Barr Virus is considered a cause of Burkitt's lymphoma and other immunosuppression related lymphomas, Hodgkin lymphoma, and nasopharyngeal cancer. Several other human herpesviruses have been linked to cancers but the totality of evidence is inconclusive.

**Methods:**

We conducted a systematic sub-study from within an ongoing case control study of adult black South Africans to investigate the relationship between antibodies to six human herpesviruses and seven cancer groups that may be caused by infectious agents. Subjects had incident cancers of the oral cavity(n = 88), the cervix(n = 53), the prostate(n = 66), Hodgkin lymphoma(n = 83), non-Hodgkin lymphoma(n = 80), multiple myeloma(n = 94) or leukaemia(n = 203). For comparison, patients with other cancers(n = 95) or cardiovascular disease(n = 101) were randomly selected from within the study. Patients were interviewed and their blood was tested for IgG antibodies against HSV-1, HSV-2, VZV, EBV-EBNA, CMV and HHV-6 using enzyme linked immunosorbent assays. Because these viruses are highly prevalent in this population, optical density results from the assays were used as an indirect, quantitative measure of antibody level.

**Results:**

There was significant variation in the mean log antibody measures for HSV-2, VZV, CMV and HHV-6 between the disease groups. However, none of the specific cancer groups had significantly higher mean log antibody measures for any of the viruses compared to either control group. In a more detailed examination of seven associations between cancers and herpesviruses for which there had been prior reports, two statistically significant associations were found: a decreasing risk of myeloid leukaemia and an increasing risk of oral cancer with increasing tertiles of antibodies against HHV-6 compared to all other patients (p-trend = 0.03 and 0.02, respectively). Odds ratios for the top tertile compared to the bottom tertile were 0.58 (95%CI 0.3 – 1.0) for myeloid leukaemia and 2.21 (95% CI 1.1 – 4.3) for oral cancer.

**Conclusion:**

In this population, using these tests for IgG, neither mean antibody measure nor high antibody measure against human herpesviruses 1–6 was strongly associated with any of the seven cancer groups. However, we may not have had sufficient power to detect weak associations or associations with a sub-type of cancer if they were present.

## Background

Infection with certain types of human herpesviruses has been established as a cause of several cancers. These include Epstein-Barr Virus (EBV) for Burkitt's lymphoma and other immunosuppression related lymphomas, Hodgkin lymphoma, and nasopharyngeal cancer [1]; and human herpesvirus 8 (HHV-8) for Kaposi's sarcoma [2]. These cancers are rare responses to the presence of these widespread viruses.

Several human herpesviruses have been linked to other cancers although the totality of evidence is inconclusive. For example herpes simplex virus type 1 (HSV-1) has been associated with oral cancer[3] and herpes simplex virus type 2 (HSV-2) with cervical cancer in women who are co-infected with specific human papillomavirus types[4]. Human herpesvirus type 6 (HHV-6) has been linked to Hodgkin lymphoma [5], acute myeloid leukaemia [6] and oral cancer [7]. In addition it has been suggested that prostate cancer [8] and multiple myeloma [9] may have infectious causes.

Our group previously found that high antibody levels to HHV8 are highly correlated to the diagnosis of Kaposi's sarcoma [2]. We therefore designed a study to examine, in a systematic way, antibody levels to six of the herpesviruses (HSV-1, HSV-2, Varicella Zoster (VZV), EBV, cytomegalovirus (CMV) and HHV-6) in relation to seven cancer groups for which there is some evidence of an infectious cause (oral, cervical, prostate, Hodgkin lymphoma, non-Hodgkin lymphoma, multiple myeloma and leukaemia). The study was part of a larger case-control study of the causes of cancer in black South Africans, which was conducted in public hospitals that treat cancer in greater Johannesburg, South Africa [2,10]. Since most human herpesviruses are highly prevalent, and PCR on biopsy samples is unrealistic in this setting, we used quantitative measures of anti-human herpesvirus antibodies from enzyme linked immunosorbent assays (ELISAs). In addition we examined the relationships between demographic and lifestyle factors and antibody levels against these viruses, as little is known about these viruses in this population.

## Methods

### Study Participants

The study population has been described previously [2,10]. Briefly, between March 1995 and February 1999 trained nurses interviewed adult black patients with newly diagnosed cancer at tertiary government hospitals in Johannesburg (Chris Hani-Baragwanath, Hillbrow, and Johannesburg General Hospitals). A standard questionnaire, administered in the language of the patient (usually an Nguni or Sotho group language), was used. Questions were asked about socio-demographic factors and behavioural characteristics including age, sex, birthplace, residence, level of education, tobacco and alcohol use, and reproductive and lifetime sexual history.

Blood was collected from 84% of patients at the time of interview and prior to commencing treatment. The remainder were too ill, had collapsed veins, or refused consent. All interviewed patients with oral cancer (n = 88), Hodgkin lymphoma (n = 83), non-Hodgkin lymphoma (n = 80), multiple myeloma (n = 94) and leukaemia (n = 203) and a random sample of subjects with cervical (n = 53) and prostate cancer (n = 66) were included in the study if they had provided a blood sample. Controls were from two groups of patients attending the same hospitals: a group of patients with other cancers (n = 95) and a group with cardiovascular diseases (n = 101). The controls were selected randomly within sex and age bands, and frequency matched to the cancer cases as a whole according to five-year age-groups and sex. Diagnoses of cancer were established, where appropriate, by histology, haematology, or cytology. The study was approved by the Committee for Research on Human Subjects (Medical) of the University of the Witwatersrand, and informed consent was obtained from all patients.

### Laboratory methods

After coagulation, specimens were centrifuged to obtain serum. The serum specimens were aliquotted and stored at -20° to -30°C. HIV testing was carried out by the Serology Laboratory of the South African Institute for Medical Research (now the National Health Laboratory Service), using commercial ELISA kits. Early testing was for HIV-1 only; later testing for both HIV-1 and HIV-2. Patients with borderline results were considered to be HIV negative.

Herpes virus antibody testing was done at the South African National Institute for Virology (now the National Institute for Communicable Diseases) from January to April 2000. Throughout the testing period specimens were kept at 4°C. The commercially available kits used were: Eurogenetics for HSV-1, HSV-2 and CMV; Clark Laboratories for VZV and EBV nuclear antigen-1(EBNA); and PanBio for HHV-6. These were selected following apreliminary study which demonstrated good quantitative performance with South African sera. The test for EBNA IgG used "recombinant EBNA-1 antigen". All other kits used unspecified antigens.

Patients' sera were randomly allocated to the 96-well microplates to minimise potential bias due to systematic differences between the plates or day on which the plates were run. Each specimen was run in a single well. There were a total of 12 plates for each assay and in general two plates were run each day. Repeatability of each assay was investigated through replicate measures on a pool of sera samples with high antibody measures against each virus. Test results were initially in optical density units which are proportional to the concentration of antibody i.e. the antibody titre. The CMV kit had a calibration curve giving final results in antibody units/ml. The remaining kits contained standards used to determine a cut-off for positivity. Results are reported as ratios to the manufacturer's cut-off value. Throughout this paper we use the general term "antibody measure" to refer to the manufacturer's result, regardless of whether it is in AU/ml or "times cut-off".

### Statistical Analysis for the control samples

There were several potential sources of variability in each assay such as antigen binding within or between plates, washing conditions between binding steps and laboratory conditions such as the temperature, humidity, and light. For each of the herpesviruses the logarithm (log) antibody measure was computed for the control pool and analysis of variance was used to estimate the magnitude of the components of variance for each of the sources of variability.

### Statistical Analysis of the Patient Data

To examine the relation between antibodies against the human herpesviruses 1–6 and cancer the mean log antibody measures for each of the nine disease groups were compared using analysis of variance. Least square mean log antibody measures were calculated for each disease group with adjustment for age, sex, HIV status, and the day and the plate on which the assay was run. Paired comparisons were conducted between each cancer group and each of the two control groups (other cancers and cardiovascular disease patients) with Bonferroni adjustments for multiple comparisons. Where positive associations had previously been found (see Introduction [1-7]), the specific hypotheses were investigated in more detail by comparing the specified cancer group to all the other patients combined with respect to tertiles of log antibody measure. Odds ratios were calculated using logistic regression with adjustment for age, sex, HIV status and the day and the plate on which the assay was run.

Since little is known about risk factors for infection with human herpesviruses in this population, we examined the relationship between antibody measure and a number of socio-demographic variables using analysis of variance with adjustment for each of these variables as well as cancer group, day of assay, and assay plate. Due to the number of comparisons that were made these analyses were examined at the 0.01 significance level.

All analyses were carried out using SAS statistical software [11].

## Results

In total 667 patients with the seven specific cancers of interest were included in the study. A further 95 patients with other types of cancer and 101 patients with cardiovascular diseases were included as control subjects. Demographic characteristics of the study members are shown in Table 1.

**Table 1:** Demographic characteristics of patients by disease group

	Cancer group																	
			
	Lip, oral cavity and pharynx		Cervix		Prostate		Hodgkin lymphoma		non-Hodgkin lymphoma		Multiple myeloma		Leukaemia		Other cancers^1^		Cardiovascular disease	
	n	(%)	n	(%)	n	(%)	n	(%)	n	(%)	n	(%)	n	(%)	n	(%)	n	(%)
Males	64	(73)	0	(0)	66	(100)	46	(55)	45	(56)	52	(55)	92	(45)	45	(47)	45	(45)
Females	24	(27)	53	(100)	0	(0)	37	(45)	35	(44)	42	(45)	111	(55)	50	(53)	56	(55)
Age <35 yrs	6	(7)	15	(28)	0	(0)	33	(40)	22	(28)	5	(5)	57	(28)	23	(24)	17	(17)
Age 35–49 yrs	17	(19)	12	(23)	6	(9)	35	(43)	25	(31)	23	(24)	54	(27)	26	(27)	29	(29)
Age 50+ yrs	65	(74)	26	(49)	60	(91)	15	(18)	33	(41)	66	(70)	92	(45)	46	(48)	55	(55)
HIV +ve	3	(3)	5	(9)	1	(2)	9	(11)	18	(23)	4	(4)	8	(4)	6	(6)	11	(11)

Total	88	(100)	53	(100)	66	(100)	83	(100)	80	(100)	94	(100)	203	(100)	95	(100)	101	(100)

^1^Includes cancers of the digestive organs, respiratory organs, bone, skin, soft tissue, breast, female and male genital organs, bladder, brain, thyroid and of unspecified sites.

The results from the analysis of the control samples suggested that for all assays (except for HSV-1 and HSV-2) the most important source of systematic variation was the day on which the plates were assayed, whereas for the HSV-1 and HSV-2 assays it was the plates themselves (data not shown). As the plate and day on which each patient's sample was tested had been recorded, variation between days and between plates was controlled for in the main analyses by adjusting for these factors.

Crude adult prevalence rates in the study, determined using the manufacturer's criteria for positivity, were: 99% for HSV-1; 59% for HSV-2 (52% in males and 68% in females); 97% for VZV; 96% for EBV (EBNA antigen); 99% for CMV; and 90% for HHV-6 (88% in males and 93% in females).

### Associations with cancer types

**Figure 1:**
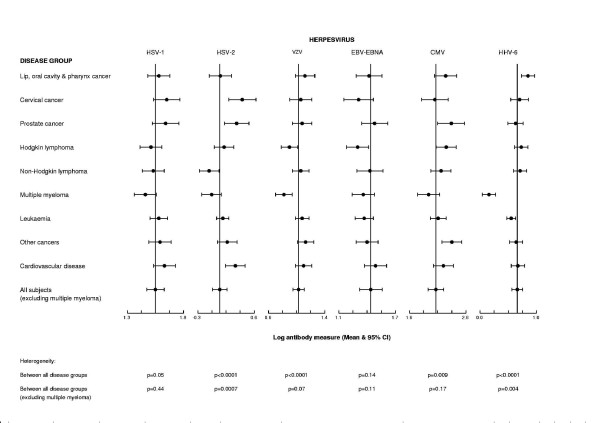
Distribution of mean log antibody measures (and 95% CI) by disease group for herpesviruses 1–6.

There was statistically significant variation between the nine disease groups in mean log antibody measure for HSV-2 (p < 0.0001), VZV (p < 0.0001), CMV (p = 0.009) and HHV-6 (p < 0.0001) (Figure 1). Patients with multiple myeloma had lower mean log antibody measures for almost all these herpesviruses compared to other patients. When patients with multiple myeloma were excluded, significant heterogeneity in mean log antibody measures between the eight remaining disease groups was still present for HSV-2 (p = 0.0007) and HHV-6 (p = 0.004).

In paired comparisons (with Bonferroni adjustments), patients with multiple myeloma had significantly lower mean log IgG antibody measures for the response to VZV, CMV and HHV-6 compared to patients in the 'other cancer' group (p < 0.0001, 0.02 and p < 0.0001 respectively) and for HSV-2, VZV and HHV-6 compared to patients with cardiovascular diseases (p = 0.01, 0.0003 and <0.0001 respectively). Because of their systematically lower antibody measures, multiple myeloma patients were excluded from further analyses. Patients with non-Hodgkin lymphoma had significantly lower mean log antibody measures in response to HSV-2 compared to the non-cancer patients (p = 0.004). None of the specific cancer groups had significantly higher mean log antibody measures for any of the viruses compared to either control group.

To further investigate the positive associations reported in previous studies (EBV and Hodgkin lymphoma; EBV and non-Hodgkin lymphoma; HSV-1 and oral cancer; HSV-2 and cervical cancer; HHV-6 with oral cancer, Hodgkin lymphoma and acute myeloid leukaemia) the log antibody measures were divided into tertiles according to the levels in allpatientsexcluding those with multiple myeloma. The control group was defined in this way for these analyses because in general mean antibody levels did not vary significantly across disease groups after exclusion of subjects with multiple myeloma (Figure 1), and including more patients increased the power of these analyses. Only oral cancer showed a statistically significant trend of risk with increasing HHV-6 tertiles (p = 0.02), odds ratio for the top tertile compared to all other subjects = 2.21 (95% CI 1.1 – 4.3) (Table 2). All the oral cancer patients were positive for HHV-6 antibodies by the manufacturer's cut-off. Only two subjects had undifferentiated nasopharyngeal cancer so it was not possible to investigate the relationship between this sub-type of oral cancers and EBV. Neither was it possible to separate acute myeloid leukaemias from chronic myeloid leukaemias with the information that was available, but there was some evidence of a decreasing trend in risk of myeloid leukaemia with increasing tertiles of HHV-6 (p = 0.03); odds ratio for the top tertile compared to all other subjects = 0.58 (95% CI 0.3 – 1.0).

**Table 2:** Odds ratios (OR) and 95% confidence intervals (CI) for specific cancers^1 ^versus all other subjects (controls)^2 ^by tertile of virus antibody measure in the controls

Virus and cancer group	Control tertiles	Cases/Controls	OR	95% CI	P-trend
HSV-1 and Oral cancer	0–5.04	23/200	1.00		
	5.05–6.07	26/199	1.06	(0.6 – 2.0)	
	6.08+	31/199	1.28	(0.7 – 2.3)	0.40
HSV-2 and Cervical cancer	0–1.08	11/91	1.00		
	1.09–2.17	11/91	0.90	(0.4 – 2.2)	
	2.18+	23/90	1.82	(0.8 – 4.1)	0.10
EBV-EBNA and non-Hodgkin lymphoma	0–3.81	24/208	1.00		
	3.82–5.29	31/203	1.32	(0.7 – 2.4)	
	5.30+	22/211	0.87	(0.4 – 1.8)	0.80
EBV-EBNA and Hodgkin lymphoma	0–3.87	33/211	1.00		
	3.88–5.31	18/208	0.49	(0.3 – 0.96)	
	5.32+	21/208	0.66	(0.3 – 1.4)	0.19
HHV-6 and Oral cancer	0–1.59	16/195	1.00		
	1.60–2.43	28/194	1.83	(0.9 – 3.6)	
	2.44+	31/195	2.21	(1.1 – 4.3)	0.02
HHV-6 and Hodgkin lymphoma	0–1.58	21/172	1.00		
(excluding oral cancers)	1.59–2.40	17/174	0.87	(0.4 – 1.7)	
	2.41+	28/172	1.32	(0.7 – 2.5)	0.39
HHV-6 and Myeloid Leukaemia	0–1.61	47/131	1.00		
(excluding oral cancers)	1.62–2.59	52/134	1.16	(0.7 – 1.9)	
	2.60+	26/131	0.58	(0.3 – 1.0)	0.03

^1^For associations that had been reported previously.

^2 ^The control group in these analyses included all other subjects, except patients with multiple myeloma, because in general mean antibody levels did not vary significantly across disease groups after exclusion of subjects with multiple myeloma (Figure 1), and including all patients increased the power of these analyses.

All analyses are adjusted for age group, sex, day of assay and assay plate.

### Association with age, sex and socio-demographic factors

Possible determinants of high antibody measures to these herpesviruses, such as age and sex and socio-demographic factors, were investigated in an analysis of variance using data from all the patients except those with multiple myeloma (Table 3). No risk factors were identified as significantly influencing high log antibody measures in response to HSV-1. For HSV-2 mean log antibody measures increased with age (p-trend = 0.003), were higher in women (p < 0.0001), increased with number of sexual partners (p-trend = 0.01) and were lower in single people (p-heterogeneity = 0.005). No statistically significant associations were found for VZV, or EBV-EBNA. Mean log antibody measure for response to CMV increased significantly with age (p-trend = 0.006) and was also higher in females than in males (p < 0.0001). Similarly, the mean log antibody measure for HHV-6 antibodies was significantly higher in females than males (p = 0.0002).

**Table 3:** Mean log antibody measure (and standard error) for human herpesviruses 1–6 according to age at diagnosis, sex and other socio-demographic factors.

Risk factor		Virus					
		
		HSV-1	HSV-2	VZV	EBV-EBNA	CMV	HHV-6
Age	<20	1.55 (0.11)	-0.19 (0.20)	1.06 (0.12)	1.52 (0.15)	1.79 (0.21)	0.64 (0.15)
	20–39	1.61 (0.04)	0.25 (0.08)	1.06 (0.05)	1.44 (0.07)	2.19 (0.12)	0.70 (0.06)
	40–59	1.61 (0.04)	0.36 (0.07)	1.07 (0.05)	1.40 (0.07)	2.26 (0.07)	0.74 (0.06)
	60+	1.60 (0.05)	0.48 (0.08)	1.11 (0.06)	1.47 (0.07)	2.41 (0.09)	0.66 (0.05)
***p-value***		***0.92***	***0.003***	***0.41***	***0.77***	***0.006***	***0.52***
Sex	Males	1.59 (0.05)	0.05 (0.08)	1.03 (0.06)	1.43 (0.07)	1.98 (0.09)	0.59 (0.06)
	Females	1.60 (0.05)	0.40 (0.08)	1.11 (0.06)	1.48 (0.07)	2.34 (0.10)	0.78 (0.06)
***p-value***		***0.91***	***<0.0001***	***0.09***	***0.44***	***<0.0001***	***0.0002***
Smoking	Never	1.60 (0.04)	0.21 (0.08)	1.03 (0.05)	1.45 (0.07)	2.16 (0.09)	0.67 (0.06)
	Ever	1.59 (0.05)	0.24 (0.09)	1.11 (0.06)	1.46 (0.08)	2.17 (0.10)	0.70 (0.06)
***p-value***		***0.91***	***0.56***	***0.07***	***0.96***	***0.94***	***0.34***
Education	< Grade 3	1.59 (0.05)	0.30 (0.09)	1.10 (0.06)	1.41 (0.08)	2.18 (0.10)	0.74 (0.07)
	Grade 3–7	1.64 (0.05)	0.24 (0.08)	1.09 (0.05)	1.47 (0.07)	2.13 (0.09)	0.69 (0.06)
	Grade 8+	1.55 (0.05)	0.14 (0.09)	1.04 (0.06)	1.49 (0.08)	2.18 (0.10)	0.64 (0.07)
***p-value***		***0.53***	***0.09***	***0.26***	***0.18***	***0.99***	***0.14***
Area of birth	Urban	1.61 (0.05)	0.28 (0.09)	1.10 (0.06)	1.46 (0.07)	2.14 (0.10)	0.70 (0.06)
	Rural	1.57 (0.04)	0.18 (0.08)	1.05 (0.05)	1.45 (0.07)	2.19 (0.09)	0.67 (0.07)
***p-value***		***0.29***	***0.13***	***0.16***	***0.67***	***0.48***	***0.45***
Area living	Urban	1.59 (0.04)	0.19 (0.07)	1.08 (0.05)	1.45 (0.07)	2.17 (0.09)	0.71 (0.06)
	Rural	1.59 (0.05)	0.27 (0.09)	1.07 (0.06)	1.46 (0.08)	2.15 (0.11)	0.67 (0.06)
***p-value***		***0.99***	***0.35***	***0.83***	***0.76***	***0.85***	***0.53***
HIV	+ve	1.53 (0.06)	0.27 (0.11)	1.12 (0.07)	1.43 (0.09)	2.22 (0.12)	0.71 (0.08)
	-ve	1.66 (0.04)	0.18 (0.07)	1.02 (0.05)	1.48 (0.06)	2.11 (0.08)	0.67 (0.05)
***p-value***		***0.03***	***0.35***	***0.10***	***0.52***	***0.34***	***0.66***
No. of	0–2	1.60 (0.05)	0.12 (0.08)	1.02 (0.06)	1.47 (0.07)	2.12 (0.09)	0.64 (0.06)
sexual	3–5	1.58 (0.05)	0.25 (0.08)	1.05 (0.06)	1.41 (0.07)	2.18 (0.09)	0.75 (0.06)
partners	6+	1.60 (0.05)	0.31 (0.09)	1.15 (0.06)	1.49 (0.08)	2.19 (0.11)	0.67 (0.07)
***p-value***		***0.96***	***0.01***	***0.03***	***0.96***	***0.34***	***0.26***
Marital	Single	1.58 (0.04)	0.06 (0.08)	1.04 (0.05)	1.45 (0.07)	2.03 (0.09)	0.65 (0.06)
status	Married	1.62 (0.05)	0.22 (0.08)	1.11 (0.05)	1.43 (0.07)	2.16 (0.09)	0.67 (0.06)
	Widowed	1.59 (0.06)	0.20 (0.11)	1.04 (0.07)	1.41 (0.09)	2.17 (0.12)	0.66 (0.08)
	Separated	1.58 (0.07)	0.42 (0.12)	1.10 (0.08)	1.54 (0.10)	2.29 (0.14)	0.77 (0.09)
***p-value***		***0.64***	***0.005***	***0.45***	***0.52***	***0.26***	***0.34***

Multivariate analysis of variance excluding patients with multiple myeloma and adjusted for all factors above plus disease group, plate and day of assay. P-values are for trend tests where appropriate and otherwise for heterogeneity.

## Discussion

Herpesvirus infections are highly prevalent in human populations. They usually produce transient illness or inapparent infection and remain latent in the host. In general, re-activation of the latent infection, re-infection, or viral persistence will cause the established IgG antibody levels to rise. In Burkitt's lymphoma it has been shown that raised levels of IgG were present months before clinical onset [12]. Antibodies to HHV-8 are also found many months prior to the diagnosis or Kaposi's sarcoma [13] and the diagnosis is highly correlated to high HHV-8 antibody levels [2]. It is therefore reasonable to speculate that raised antibody levels to other viruses may be associated with the development of other cancers.

We conducted a systematic study of the relationship between antibodies against human herpesviruses 1–6 and seven cancer groups in adult black South Africans. Our results suggest that, in this population, neither mean ELISA IgG antibody measure to the antigens used nor high antibody measures against these six human herpesviruses were strongly associated with any of the seven cancer groups. Although we do not think that the variability in the quantitative assay results concealed any strong associations, we may not have had sufficient power to detect weak associations (for example, odds ratios <2.0) or associations with a sub-type of cancer if they were present.

Though there was no evidence of a significant association between high EBV-EBNA antibody measure and non-Hodgkin lymphoma or Hodgkin lymphoma, EBV may be a causative factor of certain types of lymphomas only, including Burkitt's lymphoma and some immunosupression associated lymphomas [1]. As mentioned above, the current study was not powered to detect associations in small sub-groups. However, our results suggest that high IgG antibody measures against EBV-EBNA do not appear to be relevant in most lymphomas in this population. Several other associations between herpesviruses and cancers, which have been reported less consistently in the literature, were not clearly evident in the current study including: HSV-1 and oral cancer; HSV-2 and cervical cancer; HHV-6 and Hodgkin lymphoma; and HHV-6 and myeloid leukaemia.

There was evidence of an increased risk of oral cancer for subjects in the highest compared to lowest tertile of antibodies against HHV-6 compared to other subjects (OR = 2.21, 95% CI 1.1 – 4.3), and a trend of increasing risk across the tertiles (p = 0.02). A similar study in India of patients with Hodgkin lymphoma, non-Hodgkin lymphoma, oral cancer and healthy controls found that patients with oral cancers had elevated levels of HHV-6 antibodies compared to the healthy controls [7]. As far as we are aware no additional studies have published results on the association between HHV-6 and oral cancer, but since two studies have found a positive relationship further research is warranted. There was also an unexpected small decrease in the risk of myeloid leukaemia in patients with the highest tertile of antibodies against HHV-6 (OR = 0.58, 95% CI 0.3 – 1.0). An increased risk of acute myeloid leukaemia and HHV-6 has been reported previously in some studies [6,14,15], although other studies have not found evidence of such an association [16,17].

The retrospective design on this study means that it is possible that the cancer affected the patient's antibody levels and therefore that the antibody levels reflect a consequence rather than a cause of the cancer. There was evidence of this with respect to the multiple myeloma patients, who had lower than average mean log antibody measures for response to all six herpesviruses. This may be because myeloma is an over-production of a single immunoglobulin class, which could in turn down-regulate production of other immunoglobulins/antibodies. None of the other disease groups had a systematically higher or lower log antibody measures for response to all six of the herpesviruses. However, it remains a possibility that the positive association between raised HHV-6 antibody levels and the risk of oral cancer could have been caused by opportunistic reactivations due to cancer-associated immunosuppression. Future studies should preferably be conducted with prospectively collected blood samples.

Little is known about the determinants of infection with and antibody response to herpesviruses 1–6 in the population studied. In an analysis of associations with age, sex and lifestyle factors there was evidence that, as expected, HSV-2 antibody measures increased with increasing number of sexual partners. In addition, antibody measures to both HSV-2 and CMV were significantly higher in older age groups, presumably reflecting an increasing number of re-infections, and in women. For HHV-6, which is probably transmitted via saliva [18], antibody measures were also found to be higher in women than in men. Several other studies have reported similar findings for HHV-6 [19,20] although in the study in West Africa and the Caribbean, this was only true for children [19]. Primary HHV-6 acquisition has been found to be associated with female sex [21]. HIV positive patients in our study did not have significantly higher average antibody measures in response to any of these herpesviruses.

As quantitative methods have not previously been widely used in epidemiological studies of herpesviruses we included a quality control analysis to assess the repeatability of the assay results. We found systematic variability that could be attributed to differing laboratory conditions and for HSV-1 and HSV-2 variability attributable to either the binding of the assay components to the plates or calibration control samples that were not effective at reducing the variation between plates. For quantitative assessments improvements to the kit techniques which we used are needed, possibly using different antigens. In the current study we randomised the patients' samples to the plates so that the systematic differences will not have biased our results and took account of these systematic sources of variability in the analyses by adjusting for the plate and day on which the assay was run.

## Conclusion

In the black adult population of greater Johannesburg neither mean IgG antibody measure nor high antibody measures against human herpesvirsues 1 – 6 were strongly associated with any of the seven selected cancer groups. We do not think that the variability in the quantitative assay results concealed any strong associations, but we may not have had sufficient power to detect weak associations or associations with a sub-type of cancer. The finding of a small increased risk for oral cancer with increased antibodies to HHV-6 merits further exploration.

## Authors' contributions

VB and FS conceived of and designed the study. MU was involved in the design of the plate template for serological testing, in co-ordination of the serological testing, and in verification of patient data. NB participated in the study design, selection of test kits and design of the plate template for serological testing as well as supervision of the serological testing. MH provided pathology reports. MP, PR, and RS supplied patients for this study. AB conducted the statistical analysis and drafted the manuscript. VB, MU, RN and FS were involved in the statistical analysis and critical revision of the manuscript.
